# The impact of health system governance and policy processes on health services in Iraqi Kurdistan

**DOI:** 10.1186/1472-698X-10-14

**Published:** 2010-06-08

**Authors:** Ali Tawfik-Shukor, Hiro Khoshnaw

**Affiliations:** 1 London School of Hygiene & Tropical Medicine, London, UK; 2 Royal Surrey County Hospital NHS Trust. Surrey, UK

## Abstract

**Background:**

Relative to the amount of global attention and media coverage since the first and second Gulf Wars, very little has been published in the health services research literature regarding the state of health services in Iraq, and particularly on the semi-autonomous region of Kurdistan. Building on findings from a field visit, this paper describes the state of health services in Kurdistan, analyzes their underlying governance structures and policy processes, and their overall impact on the quality, accessibility and cost of the health system, while stressing the importance of reinvesting in public health and community-based primary care.

**Discussion:**

Very little validated, research-based data exists relating to the state of population health and health services in Kurdistan. What little evidence exists, points to a region experiencing an epidemiological polarization, with different segments of the population experiencing rapidly-diverging rates of morbidity and mortality related to different etiological patterns of communicable, non-communicable, acute and chronic illness and disease. Simply put, the rural poor suffer from malnutrition and cholera, while the urban middle and upper classes deal with issues of obesity and Type 2 diabetes. The inequity is exacerbated by a poorly governed, fragmented, unregulated, specialized and heavily privatized system, that not only leads to poor quality of care and catastrophic health expenditures, but also threatens the economic and political stability of the region. There is an urgent need to revisit and clearly define the core values and goals of a future health system, and to develop an inclusive governance and policy framework for change, towards a more equitable and effective primary care-based health system, with attention to broader social determinants of health and salutogenesis.

**Summary:**

This paper not only frames the situation in Kurdistan in terms of a human rights or special political issue of a minority population, but provides important generalizable lessons for other constituencies, highlighting the need for political action before effective public health policies can be implemented - as embodied by Rudolf Virchow, the father of European public health and pathology, in his famous quote "politics is nothing but medicine at a larger scale".

## Background

**Figure 1:**
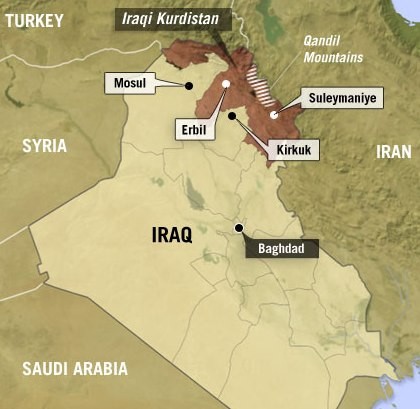
Map of Iraqi Kurdistan [Source: http://www.npr.org/

The current state of health and health services in Kurdistan (Figure 1) can be attributed to decades of successive conflict: the Iraq-Iran war (1980-88), two Gulf wars (1990 and 2003, respectively), a Kurdish civil war (1994-98) and 13 years of US sanctions (1990-03). These resulted in the collapse of infrastructure coupled with intellectual, political and socioeconomic isolation. Kurds have also been subjected to nearly a century of forced displacement and assimilation under successive Iraqi governments, culminating in the 'Anfal' genocide (1986-89) by the Baathist regime. Following an international media outcry, a No-Fly-Zone was established by the US, UK and France in 1991 to protect humanitarian operations and nearly two million Kurdish refugees fleeing the Iraqi army, stranded on the Turkish and Iranian borders [1].

**Figure 2:**
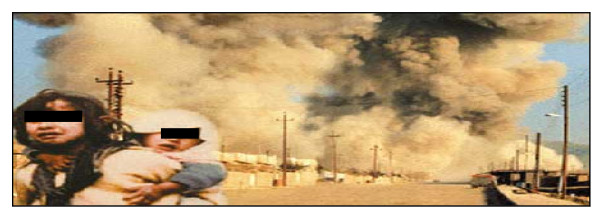
**Kurdish children fleeing Iraqi chemical weapon attack on the village of Halabja in March 1988 [Source: KRG]**.

Since 1992, Kurdistan has enjoyed relative stability and semi-autonomous status in Iraq under the No-Fly-Zone protection [2]. Despite this, Kurdistan faces a monumental challenge in the construction of its health system [3]. Under-investment, sanctions, neglect and war have taken their toll on the schools, universities, hospitals and primary care centers in the region. Thousands of people contract infectious diseases such as cholera annually due to a lack of sewage, electricity and water infrastructure [4-6]. Kurdistan also faces major challenges associated with treating long-term chronic diseases and post-traumatic mental illness resulting from successive wars, particularly the genocide and chemical weapon attacks [4,7-11]. Dozens of Kurdish villages were bombed by the Iraqi army with chemical weapons in the late 1980 s - the attack on the town of Halabja along the Iranian border left over 5000 civilians dead after exposure to mustard gas, sarin, tabun, VX and hydrogen cyanide (Figure 2).

Further exacerbating the situation, Kurdistan currently hosts the largest number of post war internally displaced Iraqis (over half a million in Sulaimani province alone), many of whom live in refugee tent camps, with little access to health care, and lacking ration cards needed to access to federal government food support [4]. The refugee crisis is further worsened by routine air raids and shelling of mountainous border villages by Iran and Turkey fighting Kurdish rebels.

## Discussion

### Health system structure & governance

**Figure 3:**
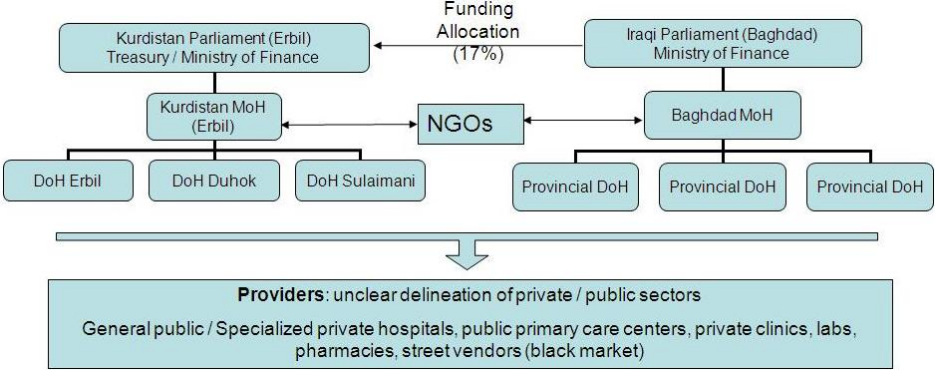
**Health system structure**.

Underlying these massive problems is a poorly designed and managed outdated health system (Figure 3). The organizational structure of Kurdistan's regional health 'system' is a microcosm of Iraq's wider national system (from which it was historically developed), reflected by its key attributes: centralized, politicized, non-transparent, disorganized, with no clear governance, regulatory, financing or accountability framework, let alone vision or goals [4]. There are two Ministries of Health (MoH) in Iraq (the Federal MoH in Baghdad, and Regional MoH in Erbil, Kurdistan), and 19 provincial Departments of Health (one in each province, and two in Baghdad) [12].

The semi-autonomous Kurdistan Regional Government (KRG) governs the provinces of Sulaimani, Erbil and Duhok (total population of 3.8 million in 2006), and has an independent Parliament and Ministry of Finance [2,11,13]. The Kurdish MoH in Erbil, is accountable to Kurdistan's Parliament (National Assembly), and funded by Kurdistan's Treasury (Figure 3). Theoretically, the KRG's budget ($9.6 billion USD for 2010) is obtained from Baghdad's Ministry of Finance (through transfer payments equivalent to 17% of oil revenues, a figure related to Kurdistan's proportion of Iraq's population) [14,15]. For the first time in the KRG's 19 year existence, the budget of the Kurdish MoH was publicly reported, amounting to around 2.5% of total public expenditure. This is comparable to the rest of Iraq, which has reportedly increased health spend from 1.3% of GDP in 2002 to 3.8% in 2006. However, accountability mechanisms to verify these figures do not exist, and the Baghdad health authorities have been reported by the US Special Inspector General for Iraq Reconstruction to be among the most corrupt [4,12].

The low priority of health is also reflected in US government and World Bank funding and policy. 2009 US spending on health, water and sanitation in Iraq account for less than 6% and 18%, respectively, of what is spent on security. The World Bank spends less than 7% of its Iraqi reconstruction budget towards healthcare programs [4]. Of this spending, very little is allocated to Kurdistan when compared to the rest of Iraq, and whatever is allocated is spent on programs irrespective of the actual need of the region (eg. spending a quarter of the World Bank health allocation on an ambulance program). In Kurdistan, what the US has to show from its $50 billion spent on Iraqi reconstruction, are around twenty (now privately run) primary care centers and a few hospital renovation projects, the majority of which are still incomplete, or of severely poor quality. USAID contractors are largely unsupervised and are not held to account for the costs incurred of quality of their work (eg. an audit of a 2006 maternity and pediatric hospital renovation project in Kurdistan found broken sewage and water systems, rendering it unusable) [4].

The relationship between Baghdad and Erbil's MoHs is characterized by standoffs rather than cooperation, with Baghdad attempting to curtail Kurdistan's autonomy by withholding or delaying funds, restricting drug supplies (many of which are either not required or near expiry, due to Iraq's inefficient federal drug clearing house) and entry of NGOs and other aid agencies [4,16]. KRG is left at the mercy of Baghdad's choices - as Baghdad decides both the volume and content of resources sent to Kurdistan, regardless of actual needs. 'In-kind' resource allocations are expected to cease in 2009, in favor of direct cash transfers, thereby increasing KRG's decision space on resource allocations.

Staff appointments at the MoH and DoHs are mainly based on political affiliation and nepotism, and not core competences, despite significant local media outcries for the establishment of a meritocracy in Kurdistan. A KRG Parliamentary health committee exists to advise Erbil's MoH, but lacks strategic planning competences and capacities. This is compounded by a severe lack of validated health indicators required for needs assessment, planning and policy (apart from some basic WHO Iraq-wide indicators, and results from subjective surveys such as the World Bank Household Socio-Economic Survey, EMRO's Iraq Mental and Family Health Surveys 2006/2007, and the Iraq Comprehensive Food Security and Vulnerability Analysis).

The general provisions of the 2010 Kurdish MoH budget are so vague, that they allow very little room for useful deliberation and discussion. What is clear is that resource allocations are based more on political whim than actual health needs matching the population, epidemiological and socioeconomic profiles of Kurdistan's constituencies. For example, Sulaimani province, the largest by population, but currently the least politically stable, receives the least resources (15.7% allocation). Impoverished rural areas receive only 27% of resources, whereas the better developed urban areas receive 73%. How the budget is operationalized, in terms of improving governance processes, developing human resources, patient records, medical procurement and improving the quality, access and cost of care are left untold. Perhaps most tellingly, over a fifth of Erbil's total resources go towards building a new office for the Ministry of Health [17].

### Primary care resources: report of a site visit

To examine the state of primary care services and interview staff, two health services researchers with backgrounds in public health made a site visit to a primary community health care center in the center of Erbil, with a catchment area of around 60,000 patients, and funded by the MoH. A wide range of human resources for health are available in the center, including primary care physicians, nurses, dermatologists, specialists, dentists and pharmacists. Facilities included a diagnostic laboratory, pharmacy, dental surgery, maternity care and an immunization clinic, none of which had the resources to function properly and provide comprehensive primary care to their constituency. There are over a dozen such primary care centers in the capital Erbil [4,12].

The functional components of the care centers require the heaviest investment, tailored to the needs of the population served. The diagnostic labs lack most basic and necessary equipment, leading to referrals to hospitals and expensive private diagnostic labs for most services. Much of the equipment available is outdated, unused or not needed. The pharmacies are poorly stocked and organized, drugs badly stored, with supplies always in shortage. The maternity and immunization clinics are little more than rooms with kitchen tables, posters and a fridge. There is no clear systematic approach to medical waste management and disposal. There are no patient records, computers, internet access, library or any meeting facilities for staff.

### Capacities and competences

Considering their hard environment and general lack of resources, physicians were keen to exhibit good technical skills, with many staff displaying high dedication to their work despite poor working conditions. Nevertheless, no mechanisms exist to ensure a minimum level of quality of care. Accreditation and licensing systems are outdated and un-enforced, with no requirements for continuous medical education [3,18-20]. No mechanisms exist to update staff with the latest developments in clinical guidelines or patient care, with no research or teaching agenda, and no explicit links to any medical universities or teaching facilities [7].

Finally, poor leadership and management competences lead to inefficient and ineffective use of resources and staff capabilities [4]. The roles, responsibilities and functions of staff are unclear and lead to internal power struggles and disputes. Inter-professional working conditions are very poor, with a lack of regard or respect for the role of nurses.

### Care processes

In theory, the current public primary care centers officially operate between 8.30 am and 12.30 pm. In practice, doctors were said to commonly show up late and leave early, sometimes with large queues of patients developing all the way outside the center. Patients pay a nominal co-pay of 250 Iraqi Dinars (ID) which is equivalent to US $0.25, and can see as many primary care physicians in one day as they wish. Consultation time is very short, (observed to average around 2 minutes), during which patients demand certain drugs or referrals to hospitals for diagnostics or treatment. No medical record is maintained, or any record of adverse events or complaints - and thus, no culture of clinical audit or any sort of quality control.

The primary care physician is supposed to play a gate-keeping function, but in reality the referral system is not enforced or adhered to. Simple conditions that can be treated at the care center (eg. diarrhea, fever) are overwhelmingly referred to hospitals, and patients' requests for drugs are generally unchallenged by physicians. There is no incentive for physicians to prescribe generics over brand-name drugs, as Western brand-name drugs are perceived by patients as being more effective than generics. Very little time is spent on advising patients on how to take prescriptions, which can also be easily obtained without a prescription from private pharmacies or street vendors selling improperly stored or expired drugs.

By around noon the care center is nearly empty, and private practices take over. Primary care physicians actually refer patients to their private evening practices, and feed off the hospitals' public diagnostic services to augment their own business. Erbil has a 'Doctor's Alley' in the ancient city center, where hundreds of doctors advertise their private unregulated services, without monitoring or scrutiny with regards to their quality, safety or cost.

The findings presented in this section were later universally acknowledged and validated by a multitude of healthcare providers, academics, policymakers and patients spoken to at a healthcare conference in Erbil. However, further studies are needed to explore nuances and differences in structures and processes of care in different primary care settings (eg. urban, rural, public and private).

### Current policy direction - privatization using public funds

The lack of a 'systems' orientation has led to a plethora of policy initiatives, that are more in substance project plans than any sort of coordinated strategic planning aligned to key health system goals. The most alarming is the development of urban private primary care centers funded from the public purse [21]. Capital costs for the construction of nearly a dozen such centers was covered by USAID, originally intended for public use [4]. However, these centers have recently been given governmental 'pilot' approval for private provision and use.

Such policy developments are alarming, especially in light of a planned regional and further national Iraqi roll-out. The use of public funds to establish and run private clinics serving the rich is fundamentally morally flawed, and would lead to massively widening the inequities in access and quality of care that already exist, and formally reinforce the already-existing two-tier system. Most importantly, it would adversely affect the long-term socioeconomic development of the country, and lead to further impoverishment of a population exposed to catastrophic health expenditures with no social welfare or universal insurance safety net [22]. Ultimately, these trends have been proven to constitute a major poverty trap, and are reminiscent of the International Monetary Fund (IMF) and World Bank's failed structural adjustment policies [23,24].

### Health system incentives and outcomes

Negative and perverse incentives exist in many aspects of healthcare in Kurdistan - from its funding, organization and management to delivery of care. Both patient demand and the supply of services are inadequately managed. In the public domain, physicians have little incentive to treat patients or promote public health initiatives, nurses are poorly paid, disrespected and inadequately trained, and patients both over-utilize the system (due to cheap co-pay), and lack trust and respect for healthcare professionals.

The key observation from the field visit was a dysfunctional and poorly performing system in dimensions of quality, access and cost [25]. In reality, there is no 'system', but a fragmented set of services being offered by a multitude of unregulated and even unlicensed providers cannibalizing off each other, with a parasitic orientation towards the use of public hospitals. In terms of quality, patients are not receiving safe, effective and patient-centered care. The patient experience is largely unsatisfactory, resulting in a lack of trust and respect in the health system and its staff.

Finally, the system is highly inefficient - the public primary care centers are little more than referral stops, with the bulk of referrals to hospitals and diagnostic labs being avoidable. The Ministry is paying for these unnecessary hospital expenses, and the public is paying a significant portion of their income for private care and brand-name drugs. Drugs dispensed virtually free by the public system are often resold on the black market.

The result of all this is that everyone pays more - the government for major organizational flaws, healthcare staff disillusioned with un-motivating jobs, and most importantly, the patients with their health.

### A governance & policy framework for change

Currently, both Baghdad and Erbil's MoHs lack clear strategic policy directions, resulting in uncoordinated planning and fragmented projects. Through consultation with key stakeholders, Erbil's MoH needs to define the core values underlying a future health system, identify their key goals, and subsequently plan and implement aligned strategic policies. The precondition is the availability of a basic set of reliable, valid, meaningful and timely indicators of health system structures, processes and outcomes on macro (governance and financing), meso (organizational) and micro (clinical delivery) governance levels. Kurdistan's MoH must rationalize and base its policies and budgetary resource allocations on evidence of health needs, taking into account their constituencies' epidemiological and socioeconomic profiles, complemented with clear operationalization plans.

Policymakers need to start developing clear and realistic evidence-informed plans related to the basic governance functions of a health system, particularly: financing, funding, regulation (eg. quality, access and cost; private vs public), infrastructure development and management, cost-effectiveness analyses of public health interventions, accountability and performance management, inter-sectoral policy action (eg. linking water, electricity, sanitation, housing, security, welfare, education, finance and employment policies), human resources for health (eg. renewing and developing health and social care programs aligned to health needs; accreditation and continuous education; integration of education, research and patient care; enhancing leadership and management competences; ethics), regulating pharmaceutical and medical device procurement and distribution, development of appropriate health and social care delivery models (particularly for disability, maternal, child, mental and rural health), integration of public health functions (health protection, promotion and disease prevention) into primary care, and the development of health information systems and public health observatories - to name a few.

It is important to stress that policies promoting the use of public funds to establish and run private unregulated clinics will greatly widen inequities in access and quality of care, and adversely affect the long-term socioeconomic development of the country.

Operationalizing and implementing concerted health policies by Kurdistan's parliament and MoH requires the strengthening and development of Kurdistan's governance system. This is a mammoth task - as there are massive planning and control deficiencies in the current system. It is critical to examine decision-making processes within Kurdistan's decentralized governance model, and to strengthen planning and management competences, along with governance and accountability, interactions between the providers, Directorates, MoH and Health Committee within Kurdistan's National Assembly [4]. The culture of accountability and feedback must also be extended to international organizations and NGOs operating in Kurdistan, to align the needs of the population with their proposed activities, and to hold them to account in the case of gross negligence or failure.

This will inevitably take years to develop, and must take into account socioeconomic, historical - and perhaps most importantly - political contingencies. Nevertheless, it is clear that the health system's vision should have a public health orientation, with a focus on community health and primary care, and aligned to the principles and recommendations of the 1978 Alma Ata Declaration and 2008 WHO Report. Such a vision has been proven to be attainable under similar circumstances, as highlighted in the Report's special section entitled "Primary health care in action: the Islamic Republic of Iran", providing insights for comparative cross-learning from Kurdistan's neighbor [26,27]. Iran's primary care reforms during the Iraq-Iran war (1980-88), involving the establishment of rural 'health houses' and training of village health workers, has now ensured that over 90% of the rural population of 23 million have access to primary care services [28]. Many of the Iranian rural villages covered by the scheme are Kurdish, and were developed in a post-conflict setting, with similar cultural and demographic characteristics to their Iraqi counterparts [29].

## Summary

Compared to the amount of media and political attention paid to Iraq since 2003, there has been a lack of interest by the international scientific research community on the health and socioeconomic impact of the war. This paper highlights the current dire state of health services of Kurdistan, which currently fails to provide an affordable, basic level of primary care to its population. The relevance of this topic is not isolated to Kurdistan in terms of a human rights or special political issue of a minority population, but provides important generalizable lessons for other developing countries, highlighting the need for bold, evidence-informed political action to improve population health, as recently reaffirmed by Mackenbach [30]. We also hope to have provided an overview that highlights the needs of a region and people neglected by the international community for too long, and to stimulate future research in areas ranging from financing, organization, funding, management and delivery of healthcare in Kurdistan.

## Abbreviations

NGOs: Non Governmental Organizations; WHO: World Health Organization; IMF: International Monetary Fund; USAID: USAgency for International Development; KRG: Kurdistan Regional Government; MoH: Ministry of Health.

## Competing interests

The authors declare that they have no competing interests.

## Authors' contributions

ATS and HK jointly conceived, developed and drafted the paper. Both authors have read and approved the final manuscript.

## Pre-publication history

The pre-publication history for this paper can be accessed here:

http://www.biomedcentral.com/1472-698X/10/14/prepub

## References

[B1] Baggs AC Kurdistan provided interesting classroom for Canadian military doctors, medical assistants Cmaj 1991 145 6 687 8 692-5 1893324 PMC1335783

[B2] Gunter MM Natali D Olson R Özcan NA Salih K Yavuz MH The Kurds in Iraq Middle East Policy 2004 11 1 106 131 10.1111/j.1061-1924.2004.00145.x

[B3] Husni M Taylor F Koye N Medical education and health care in Iraqi Kurdistan in the last four decades Med Confl Surviv 2006 22 4 292 8 10.1080/13623690600945214 17191625

[B4] Webster P Reconstruction efforts in Iraq failing health care Lancet 2009 373 9664 617 20 10.1016/S0140-6736(09)60382-2 19238694

[B5] Dyer O Aid agencies launch appeal for Iraqi refugees, while cholera spreads in northern Iraq BMJ 2007 335 7621 637 10.1136/bmj.39349.436898.DB 17901497 PMC1995465

[B6] WHO Outbreak news. Cholera, Iraq--update Wkly Epidemiol Rec 2007 82 41 357 8 17933085

[B7] Webster P Profile Affan Hamakhan Jafar: treating trauma in northern Iraq Lancet 2009 373 9664 623 10.1016/S0140-6736(09)60385-8 19231619

[B8] Ahmad A Posttraumatic stress among children in Kurdistan Acta Paediatr 2008 97 7 884 8 10.1111/j.1651-2227.2008.00873.x 18532935

[B9] Ahmad A Posttraumatic stress disorder in children after the military operation "Anfal" in Iraqi Kurdistan Eur Child Adolesc Psychiatry 2000 9 4 235 43 10.1007/s007870070026 11202098

[B10] Mohamed-Ali H [Late lesions due to poison gas in survivors of the Iraqi poison warfare against the Kurdish people] Wien Med Wochenschr 1992 142 1 8 1553823

[B11] Randal JC After Such Knowledge, What Forgiveness? My Encounters With Kurdistan 1997 Boulder, CO: Westview Press 356

[B12] EMRO W Iraq Health Systems Profile 2005 World Health Organization, Eastern Mediterranean Regional Office

[B13] KRG The Kurdistan Region: Invest in the Future Washington DC 2008

[B14] KRG Iraq Revenue Sharing Law 2007 KRG: Baghdad

[B15] IRAQdirectory (2008) Nine billion dollars is Kurdistan Budget for 2009

[B16] International Crisis Group Oil for soil: toward a grand bargain on Iraq and the Kurds in Middle East Report No 20 2008 Kirkuk/Brussels

[B17] Sabir G Reform the health system of Kurdistan 2010 http://kurdistanhealth.blogspot.com/2010/02/2010-health-budget-of-kurdistan.html [cited March 9]

[B18] Khoshnaw H Shawis T Health and Education Needs in Kurdistan BMJ Careers 2004

[B19] Amin NM Khoshnaw MQ Medical education and training in Iraq Lancet 2003 362 9392 1326 10.1016/S0140-6736(03)14580-1 14579817

[B20] Al Mosawi AJ Medical education and the physician workforce of Iraq J Contin Educ Health Prof 2008 28 2 103 5 10.1002/chp.166 18521884

[B21] BFHC Brayeti Family Health Center 2008 http://www.brayeti-fhc.org/[cited 2008 June 20]

[B22] Maciocco G From Alma Ata to the Global Fund: The History of International Health Policy Social Medicine 2008 3 1 36 48

[B23] Di M Equity impacts of neoliberal reforms: what should the policy responses be? Int J Health Serv 2007 37 4 693 709 10.2190/HS.37.4.g 18072316

[B24] Whitehead M Dahlgren G Evans T Equity and health sector reforms: can low-income countries escape the medical poverty trap? Lancet 2001 358 9284 833 6 10.1016/S0140-6736(01)05975-X 11564510

[B25] Gerber M Iraq: The socio-economic situation in the KRG administered provinces Sulaimaniyah, Erbil and Duhok 2007 Swiss Refugee Council: Bern

[B26] Macinko J Starfield B Erinosho T The impact of primary healthcare on population health in low- and middle-income countries J Ambul Care Manage 2009 32 2 150 71 19305227 10.1097/JAC.0b013e3181994221

[B27] Starfield B Shi L Macinko J Contribution of primary care to health systems and health Milbank Q 2005 83 3 457 502 10.1111/j.1468-0009.2005.00409.x 16202000 PMC2690145

[B28] WHO The World Health Report 2008: Primary Health Care - Now More Than Ever 2008 WHO: Geneva

[B29] Myers W Behringer B Olsen M Rural health in Iraqi Kurdistan J Rural Health 2005 21 1 1 2 10.1111/j.1748-0361.2005.tb00055.x 15667003

[B30] Mackenbach JP Politics is nothing but medicine at a larger scale: reflections on public health's biggest idea J Epidemiol Community Health 2009 63 3 181 4 Epub 2008 Dec 3 10.1136/jech.2008.077032 19052033

